# Galaxy HiCExplorer: a web server for reproducible Hi-C data analysis, quality control and visualization

**DOI:** 10.1093/nar/gky504

**Published:** 2018-06-13

**Authors:** Joachim Wolff, Vivek Bhardwaj, Stephan Nothjunge, Gautier Richard, Gina Renschler, Ralf Gilsbach, Thomas Manke, Rolf Backofen, Fidel Ramírez, Björn A Grüning

**Affiliations:** 1 Bioinformatics Group, Department of Computer Science, University of Freiburg, Georges-Köhler-Allee 106, 79110 Freiburg, Germany; 2 Max Planck Institute of Immunobiology and Epigenetics, Stübeweg 51, 79108 Freiburg im Breisgau; 3 Center for Biological Systems Analysis (ZBSA), University of Freiburg, Habsburgerstr. 49, 79104 Freiburg, Germany; 4 BIOSS Centre for Biological Signaling Studies, University of Freiburg, Schänzlestr. 18, 79104 Freiburg, Germany; 5 Institute of Experimental and Clinical Pharmacology and Toxicology, Faculty of Medicine, University of Freiburg, Albertstr. 25, 79104 Freiburg, Germany; 6 Faculty of Biology, University of Freiburg, Schänzlestr. 1, 79104 Freiburg, Germany; 7 IGEPP, INRA, Agrocampus Ouest, Univ Rennes, 35600 Le Rheu, France; 8 Hermann Staudinger Graduate School, University of Freiburg, Hebelstrasse 27, 79104 Freiburg, Germany

## Abstract

Galaxy HiCExplorer is a web server that facilitates the study of the 3D conformation of chromatin by allowing Hi-C data processing, analysis and visualization. With the Galaxy HiCExplorer web server, users with little bioinformatic background can perform every step of the analysis in one workflow: mapping of the raw sequence data, creation of Hi-C contact matrices, quality assessment, correction of contact matrices and identification of topological associated domains (TADs) and A/B compartments. Users can create publication ready plots of the contact matrix, A/B compartments, and TADs on a selected genomic locus, along with additional information like gene tracks or ChIP-seq signals. Galaxy HiCExplorer is freely usable at: https://hicexplorer.usegalaxy.eu and is available as a Docker container: https://github.com/deeptools/docker-galaxy-hicexplorer.

## INTRODUCTION

Chromosome conformation capture techniques are now widely used to analyse the 3D conformation of chromatin inside the nucleus across a rising number of species, tissues and experimental conditions. In particular, the Hi-C protocol (1) has helped to uncover folding principles of chromatin, demonstrating that the genome is partitioned into active and inactive compartments (called A and B) (1) and that these compartments are further subdivided into topological associated domains (TADs) (2,3). Furthermore, Hi-C has allowed identification of chromatin loops (4,5), as well as enhancer–promoter interactions (6,7) and their influence on gene expression (8,9).

However, Hi-C data processing requires tabulating hundreds of millions to billions of paired-end reads into large matrices. This poses bioinformatic challenges for efficient processing of the data and subsequent analyses. Here, we introduce Galaxy HiCExplorer, a package that aims to make Hi-C data processing, analysis and visualization available to non-bioinformaticians. Our goal is to provide a software environment able to automate the whole workflow of Hi-C data analyses from raw read mapping, filtering and correction, to the computation of topological associated domains and A/B compartments, and finally to the visualization of contact matrices, along with various other genomic features and omics data. Moreover, Galaxy HiCExplorer is easy to install, maintainable, stable and well documented. The availability of a docker container in conjunction with Bioconda (http://dx.doi.org/10.1101/207092), eliminates the need for complex software and dependency installations. Finally, HiCExplorer is transparently developed by a community of collaborators based on best practices (10) for version control, code revisions, manual and automated testing and comprehensive documentation.

## COMPREHENSIVE SERVER FOR HI-C ANALYSES

Galaxy HiCExplorer is freely available at https://hicexplorer.usegalaxy.eu as well as a Docker container: https://github.com/deeptools/docker-galaxy-hicexplorer. Galaxy HiCExplorer was designed to provide an easily accessible data-analysis environment such that biomedical researchers can focus on critical research aspects instead of dealing with terminal-based applications that are not user-friendly. It smoothly integrates the HiCExplorer analysis toolset (8) into the Galaxy scientific analysis platform to provide web-based, easy-to-use and thoroughly tested workflows that provide pipelines for the most common Hi-C data processing steps.

In contrast to other available Hi-C analysis software like HiCUP (14), HOMER (15) and TADbit (16) among others (see (17,18) for a comprehensive list of tools), Galaxy HiCExplorer provides a fully comprehensive analysis pipeline available to much broader community of researchers and is not restricted to a subset of important features. HiC-Pro (19) is one of the few packages that offers a complete pipeline; however, its visualization tools are limited and it is only available as a command line tool. Similarly, Juicer (20) offers a command line tool processing pipeline while Juicebox (21) only provides visualizations. Moreover, the integration of HiCExplorer into Galaxy offers the possibility to process and integrate other data types like ChIP-Seq or RNA-Seq into the analysis using the same interface. None of the aforementioned tools offer web server access except HiFive (22).

A strong advantage of HiCExplorer is that it can take multiple matrix data formats developed by different research groups as input. Thus, it is well integrated in the landscape of Hi-C data analysis algorithms, as Hi-C matrices can be produced by other tools and visualized with HiCExplorer. Conversely, matrices can be created with HiCExplorer and then exported to be used by other software. Currently, the Galaxy HiCExplorer supports two major formats: The HiCExplorer specific h5 format and to promote standardization of Hi-C contact matrices the cooler format (23) developed within the 4D nucleome project (24).

## GALAXY HiCExplorer TOOLS AND WORKFLOWS

**Figure 1.**
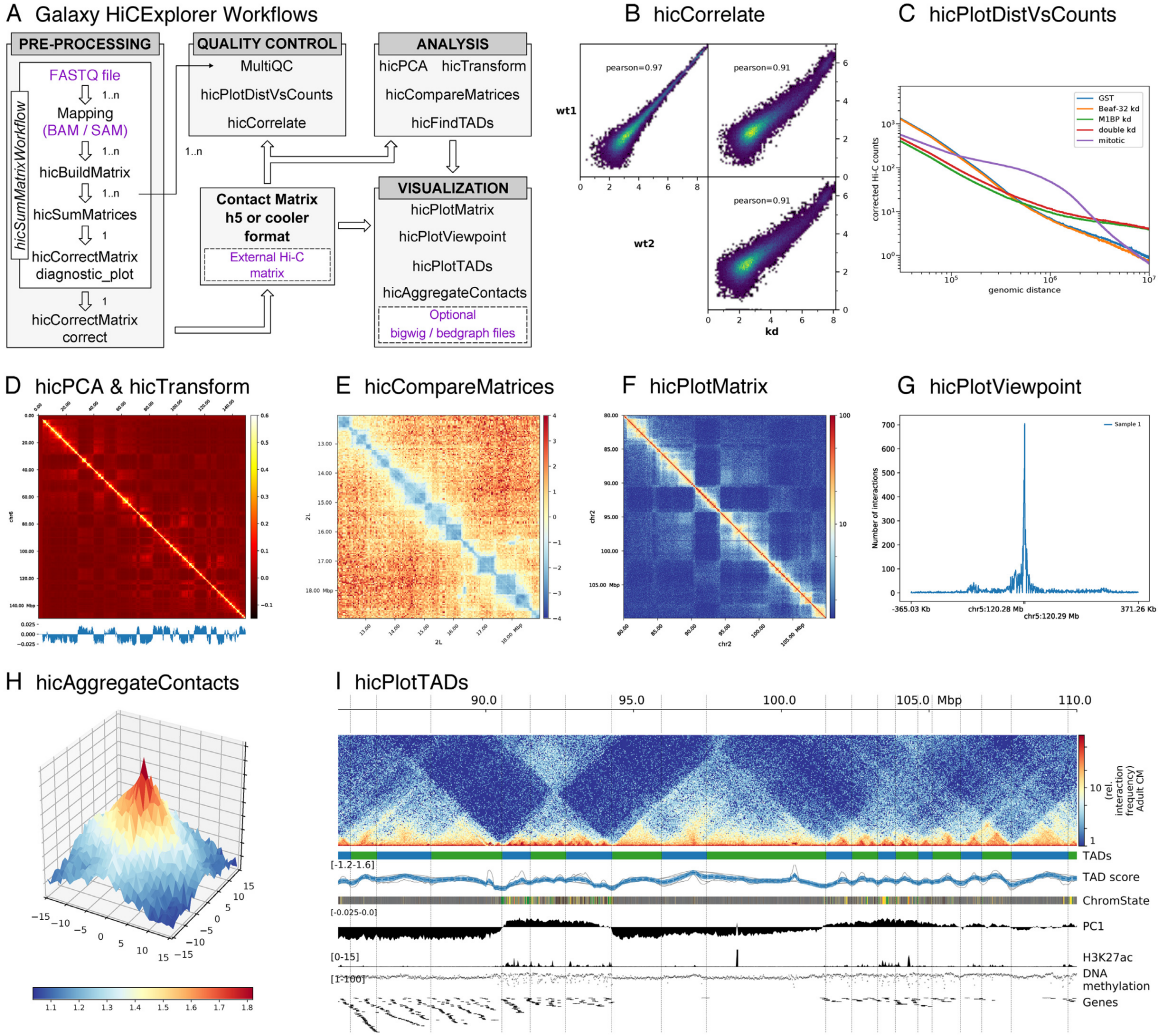
(**A**) Galaxy HiCExplorer workflows and tools. Entry points for external data are highlighted in purple. **Quality control tools:** (**B**) Output of *hicCorrelate* comparing two wild types and one knockdown samples. (**C**) Output of *hicPlotDistVsCounts* that shows changes of the number of contacts for different conditions. **Analysis tools:** (**D**) *hicPlotMatrix* of the Pearson correlation matrix derived from a contact matrix for chromosome 6 in mouse computed with hicTransform. The optional data track at the bottom shows the first eigenvector for A/B compartment obtained using *hicPCA*. (**E**) The pixel difference between a Hi-C corrected matrix for wild type condition and a knock down was computed using *hicCompareMatrices* and a 7Mb region is visualized using *hicPlotMatrix*. **Visualization tools:** (**F**) Contact matrix plot of a 80 to 105 Mb region of chromosome 2 in log scale. (**G**) Example output of *hicPlotViewpoint* showing the corrected number of Hi-C contacts for a single bin in chromosome 5 (output similar to 4C-seq) (11). (**H**) A Hi-C matrix was converted into an observed vs. expected matrix using *hicTransform* and this matrix, together with the location of high-affinity sites from (12) were used to run *hicAggregateContacts*. (**I**) 85 Mb to 110 Mb region from human chromosome 2 visualized using *hicPlotTADs*. TADs were computed by *hicFindTADs*. The additional tracks added correspond to: TAD- separation score (as reported by *hicFindTADs*), chromatin state, principal component 1 (A/B compartment) computed using *hicPCA*, ChIP-seq coverage for the H3K27ac mark, DNA methylation, and a gene track. Hi-C data for B, C, E and H from *Drosophila melanogaster* S2 cells from (8). Hi-C data for D, F and I from mouse cardiac myocytes (13). Additional tracks in I from (13).

Galaxy HiCExplorer provides a plethora of tools for processing, normalization, analysis, and visualization of Hi-C data (Figure 1A). Apart from HiCExplorer, the https://hicexplorer.usegalaxy.eu website and the Docker container also include the genome alignment tools BWA-MEM (25) and Bowtie2 (26), as well as additional tools for text manipulation, data import and quality control. The inclusion of deepTools (27) further facilitates the integration of ChIP-seq, RNA-seq, MNase-seq as well as other kind of datasets with Hi-C data.

The analysis of Hi-C data can be divided into three steps: pre-processing (including quality control), analysis and visualization.

### Pre-processing and quality control

#### hicBuildMatrix

A contact matrix is the main data structure of Hi-C data analysis which is generated from the individual alignment of valid Hi-C paired-end reads. This tool filters out potentially erroneous reads, such as unmappable reads, self-ligated reads, dangling-ends, PCR duplicates or incomplete digestions (4,14) and tabulates the results based on user defined bins (either based on restriction sites or on fixed size bins). Because building the Hi-C matrix is one of the most time consuming steps in the Hi-C workflow, we developed *hicBuildMatrix* to be multi-processing to significantly reduce running time. A comprehensive quality report is generated as an HTML file. This report includes a number of useful quality measures including: number of valid Hi-C read pairs and the number of filtered reads per category (unmappable and non-unique pairs, duplicates, dangling ends, self-circles, etc.), number of intra-chromosomal, short-range (<20 kb) and long-range contacts, and read pair orientation. Reports from multiple samples can be integrated using MultiQC (28) or using the HiCExplorer tool *hicQC*. Inspection of the *hicBuildMatrix* quality reports helps to identify potential biases or errors in the Hi-C library preparation. For example, a high number of dangling ends is indicative of a problem with the re-ligation step or inefficient removal of dangling ends. The quality report can also be useful to identify differences (long-range versus short-range contacts enrichment for instance) between samples obtained in different conditions.

#### hicMergeMatrixBins

After a Hi-C contact matrix has been created, lower resolution matrices can be obtained by merging neighboring bins. This is mostly useful for visualization at different zoom levels or to create matrices of lower resolution (larger bin size) in the event of a Hi-C matrix being too poor due to low sequencing depth.

#### hicCorrelate

This tool computes the correlation between several Hi-C matrices (Figure 1B). *hicCorrelate* can produce a scatter plot or a heatmap using either Pearson or Spearman correlations. The computation of the correlation can be restricted to a range of genomic distances to avoid biasing the correlation results with background contacts. These correlations are useful as a quality control step to compare replicates and to test for differences between various treatments.

#### hicPlotDistVsCounts

This tool plots the average number of Hi-C contacts at different genomic distances (Figure 1C). It allows the estimation of long-range and short-range contacts from multiple samples at once, and is a useful tool for both quality control and comparison of, for example, treated versus untreated samples that alter chromosome conformation.

#### hicSumMatrices

After different replicates or similarly obtained Hi-C matrices have been compared using *hicCorrelate*, they can be added up into one single contact matrix with this tool.

#### hicCorrectMatrix

Allows the removal of biases from the Hi-C matrix using a very fast version of the iterative correction algorithm from Imakaev *et al*. (29). Before the contact matrix is corrected, the right thresholds to prune values need to be selected. The *diagnostic plot* helps users in determining these thresholds.

### Analysis

#### hicFindTADs

This utility can identify TADs from a given corrected contact matrix by first computing a TAD-separation score and then identifying local minima indicative of TAD boundaries (8). In contrast to other TAD identification methods, this tool also returns the TAD-separation score, which can be visualized in a genome browser or using *hicPlotTADs*. The TAD-separation score contains useful information to identify strong and weak boundaries and the density of contacts within TAD and can be visualized along with the Hi-C matrix (see *hicPlotTADs* tool).

#### hicPCA

A/B compartments (1) refer to open and closed chromatin that is spatially separated in the cell nucleus (30,31). We compute this using eigenvector decomposition as described by Lieberman-Aiden (1) and using the first and second eigenvector. The positive/negative values correspond to open/closed chromatin. A visualization of A/B compartments is shown in Figure 1D.

#### hicTransform

The three matrices used to compute the A/B compartments (observed/expected, Pearson correlation and covariance matrices) are useful during visualization to achieve a better understanding of the Hi-C data. To enable this, *hicTransform* can compute these three matrices independently of *hicPCA*, and the matrices can then be plotted using the visualization tools.

#### hicCompareMatrices

*hicCompareMatrices* allows the computation of difference, ratio or log2ratio between two matrices. This is useful to compare replicates or samples from different conditions. It can, for example, help to characterize TAD structure modifications when followed by *hicPlotMatrix* (Figure 1E).

### Visualization

#### hicPlotMatrix

This tool is used to plot contact matrices for a collection of individual chromosomes. It has multiple options to select the matrix colors and the values range. Additionally, *bigwig* tracks can be attached to plot additional features such as A/B compartments or ChIP-seq data. It is possible to plot a multitude of domains; the entire interaction matrix, individual chromosomes, multiple chromosomes, and various regions of interest (see Figure 1D–F).

#### hicPlotViewpoint

The viewpoint plot supports a visualization of the number of interactions around a specific reference point or region in the genome, and makes the long-range interactions visible as shown in Figure 1G. The output is comparable to what is obtained using the 4C-seq protocol.

#### hicAggreateContacts

Facilitates the analysis of long range-contacts by visualizing the average contacts over multiple smaller matrices around a given set of regions (Figure 1H).

#### hicPlotTADs

To visualize the computed TADs this tool flips the main diagonal of the Hi-C contact matrix by 45° and marks the TADs with triangles. It is possible to plot multiple matrices and add additional data like genes, chromatin states, long-range interactions and any other feature that can be represented as a bigwig or bedgraph file like methylation data, ChIP-seq, or RNA-seq to visually correlate them with TADs and their boundaries. There are multiple options to select the Hi-C matrix layout and colormap, different ways to visualize genes and regions files and also multiple configurations to plot coverage tracks like color, line width, line type, as dots, filled etc. (Figure 1I).

### Workflows

Galaxy HiCExplorer provides pre-defined workflows to reduce intermediate steps and to guide a researcher through the different stages. The Galaxy framework offers the possibility to connect tools into workflows called Galaxy workflows. The provided workflows are subdivided into categories depending on the start of the analysis: First, raw FASTQ files are mapped to generate a contact matrix and its corrected equivalent. Different workflows are provided to cover the case of running many analyses in parallel or whether replicates should be merged to one contact matrix. Second, said contact matrix (or other) is used to compute TADs, A/B compartments and/or to plot them using the provided workflows. All workflows are linked on the homepage of the Galaxy HiCExplorer.

All Galaxy Workflows share a common notion that they should guide the researcher through the analysis, i.e. most parameters in the workflows do not need to be changed. The reference genome needs to be set for the mappers, and a desired bin size as well as the used restriction sites needs to be selected in order to build the contact matrix. Every workflow containing a plotting step needs the region to plot as input.

## IMPLEMENTATION

Galaxy HiCExplorer is implemented as a Docker container based on the web-based Galaxy scientific workflow platform (32). HiCExplorer itself is implemented in Python, supporting version 2.7, 3.5 and 3.6, and available as a Bioconda package (http://dx.doi.org/10.1101/207092) and as BioContainer (33). This guarantees a fixation of versions and therefore reproducibility of analysis. Galaxy wrappers for HiCExplorer are available at the Galaxy tool shed.

## USING HiCExplorer

### Installation and usage

The Galaxy HiCExplorer web server can be used by visiting http://hicexplorer.usegalaxy.eu, or by installing it on a personal computer or locally (e.g. an institute intranet). For this, pre-configured Docker containers and conda packages are available.

**Galaxy HiCExplorer:**

***Docker***:

*docker run -p 8080:80 quay.io/bgruening/galaxy-hicexplorer*

***hicexplorer.usegalaxy.eu***: On https://hicexplorer.usegalaxy.eu all HiCExplorer tools and workflows are installed. Use this option if you require high computational resources (e.g. large memory requirements).

**HiCExplorer:**

The HiCExplorer as a command line tool is available via *conda* or *BioContainers*.

***Conda***: *conda install hicexplorer -c bioconda*

***BioContainer***:

*docker run quay.io/biocontainers/hicexplorer:latest*

### Training

Training and a documentation are crucial to enable as many scientists as possible to use and understand the Galaxy HiCExplorer. To introduce scientists who are new to Galaxy a guided tour through the Galaxy interface is provided as well as a tour to learn Hi-C data analysis. The tour content is available on the Galaxy Training Network (http://dx.doi.org/10.1101/225680) as well and includes example data hosted on Zenodo. All intermediate files are available in the shared data library of the Galaxy HiCExplorer.

For advanced users a detailed step-by-step tutorial for the analysis of Hi-C data from mouse embryonic stem-cells, as well as a comprehensive API documentation, is hosted at https://hicexplorer.readthedocs.org. The how-to describes how to set up the mapping of the reads. It suggests parameter settings for the creation of Hi-C contact matrices and describes the process of merging and threshold determination to remove poor bins prior to correction. The determination of TADs using the separation score is described in detail, including examples on visualization.

## DISCUSSION

Galaxy HiCExplorer gives researchers the opportunity to run their Hi-C data analysis in a user-friendly, web browser based environment. The highly configurable framework provided by Galaxy makes this web server extendable to the various needs of researchers. Especially in conjunction with software for other high-throughput analysis protocols like RNA-seq or ChIP-seq, Galaxy HiCExplorer serves as a powerful basis for flexible explorative biomedical research in a high-throughput sequencing data analysis environment.

By combining all the necessary stages of pre-processing and visualization into a single tool, analysis not only becomes easier, but faster, highly reproducible, and more readily exchangeable. Biomedical researchers can focus their efforts on their data analysis without having to concern themselves with the particulars of managing various different software setups and configurations or learning to use command-line tools in an UNIX environment.
